# Role of Ethics in Developing AI-Based Applications in Medicine: Insights From Expert Interviews and Discussion of Implications

**DOI:** 10.2196/51204

**Published:** 2024-01-12

**Authors:** Lukas Weidener, Michael Fischer

**Affiliations:** 1 Research Unit for Quality and Ethics in Health Care UMIT TIROL – Private University for Health Sciences and Health Technology Hall in Tirol Austria

**Keywords:** artificial intelligence, AI, medicine, ethics, expert interviews, AI development, AI ethics

## Abstract

**Background:**

The integration of artificial intelligence (AI)–based applications in the medical field has increased significantly, offering potential improvements in patient care and diagnostics. However, alongside these advancements, there is growing concern about ethical considerations, such as bias, informed consent, and trust in the development of these technologies.

**Objective:**

This study aims to assess the role of ethics in the development of AI-based applications in medicine. Furthermore, this study focuses on the potential consequences of neglecting ethical considerations in AI development, particularly their impact on patients and physicians.

**Methods:**

Qualitative content analysis was used to analyze the responses from expert interviews. Experts were selected based on their involvement in the research or practical development of AI-based applications in medicine for at least 5 years, leading to the inclusion of 7 experts in the study.

**Results:**

The analysis revealed 3 main categories and 7 subcategories reflecting a wide range of views on the role of ethics in AI development. This variance underscores the subjectivity and complexity of integrating ethics into the development of AI in medicine. Although some experts view ethics as fundamental, others prioritize performance and efficiency, with some perceiving ethics as potential obstacles to technological progress. This dichotomy of perspectives clearly emphasizes the subjectivity and complexity surrounding the role of ethics in AI development, reflecting the inherent multifaceted nature of this issue.

**Conclusions:**

Despite the methodological limitations impacting the generalizability of the results, this study underscores the critical importance of consistent and integrated ethical considerations in AI development for medical applications. It advocates further research into effective strategies for ethical AI development, emphasizing the need for transparent and responsible practices, consideration of diverse data sources, physician training, and the establishment of comprehensive ethical and legal frameworks.

## Introduction

### Background

Artificial intelligence (AI) has been considered a key technology in medical advancement for several years [1]. Recent developments in AI, exemplified by the broad availability and widespread use of advanced AI-based chat applications, such as ChatGPT, have underscored the capabilities of technology [2]. This study specifically focuses on AI-based applications in medicine, highlighting the importance of ethics in their development, with an emphasis on the role of developers. Considering the inherent complexities associated with AI and its applications in medicine along with the multifaceted nature of AI ethics, this introduction aims to provide a comprehensive foundation for this publication.

### Artificial Intelligence

Early definitions of AI, such as by McCarthy et al [3], primarily focused on the potential for machines to simulate all facets of human intelligence: *“...*the basis of the conjecture that every aspect of learning or any other feature of intelligence can in principle be so precisely described that a machine can be made to simulate it.” Newer definitions, such as the one from the European Parliament, expand this scope and describe AI as “the ability of a machine to display a range of humanlike capabilities, including reasoning, learning, planning, and creativity*,*” encompassing a broader spectrum of intelligent behaviors [4].

Following the evolving definitions of AI, the term broadly encompasses various technologies, each with unique characteristics and applications. The scientific community commonly categorizes these technologies as “strong AI” and “weak AI” [5]. “Strong AI” refers to systems whose cognitive capabilities are comparable with human intelligence across a wide range of tasks and contexts [5]. However, most current applications, particularly in medicine, are categorized as “weak AI.” This category includes systems designed to perform specific tasks using cognitive abilities comparable with those of humans but within a limited scope [6]. Within the category of “weak AI,” 2 primary subfields are prominent: expert systems and machine learning (ML) [6]. Expert systems, categorized under “symbolic AI,” operate based on predefined rules and instructions set by human experts [7]. In contrast, ML represents the “statistical AI” subfield [8]. ML focuses on pattern recognition within large data sets, enabling the system to learn and make predictions or decisions based on the data [9]. A notable example of such advancements in “statistical AI” is the development of large language models, such as ChatGPT, which demonstrate the evolving capabilities of AI in understanding and generating humanlike text, offering new possibilities, and raising unique ethical considerations in their application [10].

Despite the significant technological advances in the field of AI and, in particular, “weak AI,” “strong AI,” which would entail cognitive abilities on par with human intelligence across diverse areas, remains largely theoretical with no substantial application in medicine to date [11]. Therefore, “weak AI” will be the foundation of this publication, specifically focusing on the development and associated ethical considerations of “symbolic AI” and “statistical AI” applications in medicine.

### AI in Medicine

The technological advancements and capabilities of AI in medicine, as exemplified by a range of AI-based applications such as ML algorithms and expert systems, are anticipated to transform various aspects of health care, such as diagnostics or personalized treatment planning [1].

For example, ML algorithms, a key subset of “statistical AI,” are of particular interest in medicine because of their capability to analyze large data sets, including a wide array of medical images such as x-rays, magnetic resonance imaging, computed tomography, and dermatological photographs [8]. In radiology, ML algorithms enhance image interpretation by identifying the features associated with specific pathologies. For instance, in mammography, ML assists radiologists in detecting microcalcifications and subtle changes in the breast tissue, which may indicate the early stages of breast cancer [12]. Similarly, in dermatology, ML-powered tools analyze photographic data of skin lesions and moles, thereby providing critical diagnostic insights [13]. By distinguishing between benign and malignant lesions with high accuracy, the early detection of skin cancer can be improved. The integration of ML in image-based diagnostics can not only enhance diagnostic accuracy but also have the potential to speed up the diagnostic process [8]. This reduction in analysis time leads to quicker diagnostic outcomes, enabling earlier intervention and treatment, which are crucial for improving patient care [14].

Expert systems in medicine, a subfield of “symbolic AI,” are primarily exemplified by Clinical Decision Support Systems (CDSS) [15]. By leveraging predefined rules and knowledge from medical experts, these systems can provide recommendations for diagnosis and therapy options, potentially enhancing the decision-making process in clinical settings [16]. CDSS often use information from various sources, such as electronic health records, patient history, and latest medical research, to offer evidence-based suggestions. In addition to offering diagnostic and treatment guidance, CDSS can play a significant role in identifying potential adverse drug events, which is a critical aspect of patient safety [16]. By cross-referencing a patient’s current medications with the proposed treatments, CDSS can alert health care providers to possible drug-drug interactions, allergic reactions, or contraindications based on the patient’s medical history or known conditions [15].

In addition to diagnostic and decision support applications, AI contributes to other areas of medicine, such as medical research and drug development. In medical research, AI algorithms are used to analyze complex information, such as genetic, environmental, and lifestyle data, which can be used for personalized medical approaches, enabling more targeted therapies based on individual patient profiles [17]. Furthermore, AI can be used to identify potential therapeutic compounds more quickly and efficiently than traditional methods [18]. AI systems can simulate and predict how different compounds interact with biological targets, thereby reducing the time and cost of drug trials. This capability is particularly crucial in rapidly responding to emerging global health challenges, such as the development of vaccines and treatments for new diseases [18]. Furthermore, although AI-based chat applications, such as ChatGPT, have not been specifically developed for use in medicine, they possess extensive medical knowledge, making their potential application in various medical contexts a subject of increasing interest [2]. Although advancements in the field of AI can offer transformative benefits for medicine, they also introduce new ethical considerations and challenges that warrant attention [19,20].

### AI Ethics

AI ethics can be defined as “a set of values, principles, and techniques that employ widely accepted standards of right and wrong to guide moral conduct in the development and use of AI technologies*”* [21]. Although this definition does not specifically focus on or include the field of medicine, it emphasizes the importance of values and principles in the development of AI technologies. In medicine, the fundamental principles of medical ethics formulated by Beauchamp and Childress—autonomy, nonmaleficence, beneficence, and justice—are of paramount influence and relevance [22].

The principle of autonomy emphasizes respecting patients’ rights to make informed decisions regarding their own health. In the context of AI-based applications in medicine, the principle of autonomy often refers to the development of technologies that support and enhance patient decision-making while maintaining transparency, explainability, and accountability [23,24]. This also refers to the development of AI-based applications that not only provide accurate diagnostic and treatment recommendations but also present their findings in a manner that is understandable and useful for both patients and health care professionals. The principle of nonmaleficence, emphasizing the commitment to do no harm, has become increasingly important in the context of growing role of AI in health care. Adhering to this principle requires the establishment of stringent safety protocols and comprehensive testing of AI technologies to prevent unintended consequences, such as biases in decision-making that could lead to misdiagnosis or unequal treatment of patients [24].

Bias in AI systems, particularly in medical applications, is a significant concern. For instance, ML algorithms used in image-based diagnostics, such as those used in radiology or dermatology, may develop biases based on the data they are trained on [25]. If these algorithms are primarily trained on data sets that lack diversity, they might be less accurate in diagnosing conditions in patient populations that are underrepresented in the training data [26]. This can lead to disparities in diagnostic accuracy and effectiveness, potentially harming certain groups of patients [16,27]. Similarly, in CDSS, which rely on predefined rules and medical knowledge, there is a risk of inherent biases being transferred into the system. If the input data or rules within these systems reflect historical biases or unequal treatment practices, the CDSS might perpetuate these issues, leading to recommendations that are not equitable or appropriate for all patients [16].

Addressing the challenges related to autonomy and nonmaleficence is fundamental for ensuring that AI in medicine aligns with the principles of beneficence and justice. The principle of beneficence, or acting in the best interests of the patient, emphasizes that AI-based applications in medicine should be developed with the primary goal of improving patient outcomes and enhancing quality of care [23]. Finally, the principle of justice requires that AI technologies in health care promote fairness and equity. This means ensuring equitable access to the benefits of AI advancements regardless of a patient’s socioeconomic status or background [24].

In light of these ethical principles, the role of developers in creating AI-based applications in medicine has become critically important. Developers bear a particular responsibility to ensure that the design and implementation of these technologies adhere to the ethical standards outlined by autonomy, nonmaleficence, beneficence, and justice [28]. A deep understanding and awareness of the ethical implications during the development process are essential, as the principles and guidelines frequently discussed in the current literature should be integrated from the early stages of AI application development [29,30]. This integration is not just theoretical but requires practical implementation and consistent consideration throughout the development process of AI-based applications in health care [31]. Despite the crucial role that developers play in embedding these ethical principles into AI technologies, there remains a gap in the literature regarding how developers perceive and prioritize ethics in their work [32,33]. Addressing this gap is essential for ensuring the responsible development and use of AI in medicine and aligning technological advancements with the core values of medical ethics.

### Objective

The field of AI-based medical applications is rapidly advancing; however, a significant gap remains in understanding how ethical considerations are integrated into this development process. Recognizing the frequent calls in the literature for consistent inclusion of ethics in AI development, this study aimed to bridge this gap by exploring the perceptions, priorities, and conflicts related to ethics among AI experts. Specifically, this study sought to answer the following questions:

- How do AI experts perceive the role of ethics in the development of AI-based medical applications?
- How do AI experts perceive the relationship between ethical considerations and the technical development of AI-based applications in medicine?

The primary objective of this study is not only to answer these critical questions but also to provide an in-depth discussion of the results, particularly focusing on the associated ethical implications. This exploration is vital for understanding how ethical considerations can be more effectively integrated into the development of AI technologies in medical settings with the aim of contributing to the responsible and beneficial advancement of this field.

## Methods

To address the study’s objective, a secondary analysis of the exploratory expert interviews was performed using qualitative content analysis. These interviews were initially conducted to explore the essential knowledge and understanding of AI in medicine, with the aim of specifying teaching content on AI for medical education [34].

### Ethical Considerations

Ethics approval was granted by the Research Committee for Scientific Ethical Questions of the UMIT TIROL—Private University for Health Sciences and Health Technology, Hall in Tirol, Austria, for both the initial data collection and secondary analysis of the data relevant to this study (approval number: 3181; January 16, 2023).

The methodology and reporting of the research findings in this study were guided by the Standards for Reporting Qualitative Research to ensure clarity and transparency [35].

### Expert Characteristics

Of the 12 experts included in the primary research study, 7 met the inclusion criteria for this study and provided information relevant to the study objective. For this secondary analysis, individuals were defined as experts if they had been engaged in the research or practical development of AI-based applications in medicine for at least 5 years. In this regard, 4 experts were involved in the development of AI-based applications as part of their research activities (eg, researchers at the German Research Center for Artificial Intelligence, professor for medical informatics), such as enhanced AI-assisted imaging. The remaining 3 experts were primarily engaged in the practical development of various AI-based applications for use in medicine (eg, voice recognition in hospitals or assistance in diagnosis in medical practices) as part of their main professional activities in the private sector (eg, software development). Additional inclusion criteria were sufficient language skills (German) and consent for the transcription of the interviews and their evaluation. All 7 participating experts were situated and working in Germany, providing a national perspective on the development of AI in medicine. Of the 7 experts included in this secondary analysis, 6 identified as male and 1 (E2) identified as female. Although all experts met the inclusion criteria of being engaged in research or the practical development of AI-based applications in medicine for at least 5 years, 3 experts (E1, E2, and E4) had more than 10 years of professional experience in the relevant field. In addition, 3 experts had more than 15 years of experience in the field of research and practical development of AI-based applications (E3, E5, and E7). Table 1 presents a detailed overview of the experts’ characteristics included in this study.

**Table 1:** Characteristics of the experts included in the secondary analysis.

Expert number	Professional position	Domain of expertise
E1	Research and development (AI^a^)	Machine learning in pathology
E2	Data scientist	AI in radiology
E3	Senior software developer	Clinical Decision Support Systems
E4	Research and development (AI)	AI in cancer diagnosis
E5	Professor for medical informatics	Natural language processing in medicine
E6	Data scientist	AI-assisted voice analysis for diagnosis
E7	Senior software developer	Clinical Decision Support Systems

^a^AI: artificial intelligence.

### Data Collection

In the initial data collection phase of the primary study, experts were recruited primarily via email. In addition, participants were asked to recommend other potential experts for the interviews, thereby expanding the recruitment network. This direct recommendation approach enabled the inclusion of 2 additional experts in the primary study. Before the interviews were recorded, the experts were informed about the study and the associated data protection regulations during recruitment and at the beginning of the interviews. All interviews were conducted using a video service provider (Cisco Webex Meetings) and were recorded on an audio basis (manual recording via an analog dictation device; average interview length 34.02, SD 4.1 minutes).

To ensure the protection of all collected and generated data, they were stored offline on a password-protected storage device in a lockable cabinet, with access limited to the researcher. The anonymized data will be stored for 10 years following the date of collection to enable reproducibility and deleted after to ensure confidentiality. All participating experts explicitly consented to both the initial analysis and the use of their data for future research purposes, as in the case of this study.

### Data Analysis

The expert interviews were transcribed using the transcription software f4transcript and anonymized according to the transcription rules of Dresing and Pehl [36]. The evaluation of the collected data was conducted with software support (QCAmap, version 1.2.0; Microsoft Excel, version 16.66) and was rule based according to the methodology of qualitative content analysis by Mayring (inductive procedure) [37]. Relevant categories were defined directly from the material and were controlled or revised after viewing 40% of the material. After defining the categories, the entire material was reviewed, and relevant text passages were assigned to the respective main and subcategories.

The interviews were conducted and analyzed in German. For this publication, all identified and relevant text passages were translated into the English language. The primary research team conducted the initial translation, followed by a review and revision by a professional academic translator.

It is noteworthy that the data analysis in this study was guided by the research team’s perspective and understanding of ethics. As such, the interpretation of the data and subsequent conclusions are shaped by the team’s affiliation with the research unit for quality and ethics in health care. Consequently, ethical considerations, particularly in health care and medicine as well as in the development and application of AI technologies in these fields, are considered important. The emphasis on ethics should be considered when interpreting the results of this study.

Furthermore, the aspect of theoretical saturation in this secondary analysis warrants detailed discussion. Given its distinct objectives, this study selectively used interviews with 7 of the 12 experts, chosen based on the specific inclusion criteria of engagement in research or practical development of AI-based applications in medicine for over 5 years. The remaining 5 experts from the primary study, who primarily focused on teaching and research without a direct emphasis on developing AI-based applications for medicine, did not meet the inclusion criteria for this secondary analysis. This selection, inherent to the secondary nature of the data, led to a focused but relatively limited breadth in certain areas, resulting in incomplete saturation in the 2 subcategories. Specifically, the subcategories of “Data Protection” (section *Subcategory 3: Data Protection*) and “Demands” (section *Subcategory 3: Data Protection*) demonstrated incomplete saturation, each substantiated by only a single reference. In contrast, theoretical saturation for the other categories can be assumed, given the multiple references that support the established themes and the lack of new insights, suggesting the need for additional categories.

Acknowledging this limitation is crucial, particularly in the context of future research opportunities aimed at more comprehensively exploring these underrepresented areas. However, the reliability of the results extends beyond the theoretical saturation. It is also underscored by the expertise and extensive experience of the participating experts, each with at least 5 years of AI research or practical development in medicine. Their profound insights, combined with the systematic and iterative analysis methodology, ensured that the extracted themes were representative and comprehensive, despite the gaps noted in certain subcategories. Consequently, although the findings in the “Data Protection” and “Demands” categories might benefit from further exploration in future studies, the current analysis offers a robust and insightful understanding of the primary themes related to ethical considerations in AI development for medical applications.

To ensure detailed and comprehensive data collection, a semistructured interview guideline was used for primary data collection. This interview guideline included questions directly related to the study’s objectives and incorporated both immanent and exmanent questioning. Reflecting the research team’s focus on ethics in health care and medicine, the semistructured interview guidelines incorporated 2 questions directly relevant to the study’s objectives:

- How do you perceive the role of ethics in the context of AI-based medical applications?
- What are your experiences with ethical considerations and the development of AI-based applications in medicine?

In addition to the 2 questions directly addressing the objective of this study, an interview guideline was constructed to promote openness by emphasizing the immanent and exmanent questions. Examples of the questions used are as follows:

- You have mentioned the challenge of integrating ethics into AI development. Could you elaborate on the specific ethical considerations you find most relevant in this context?
- In your view, who should bear responsibility for the ethical issues in AI-based applications—users or developers?

Using both direct and immanent as well as exmanent question types, the interviews aimed to provide an in-depth exploration of the topic of AI in medicine, including the development of AI-based applications for use in medicine.

## Results

### Overview

On the basis of the qualitative content analysis of the expert interviews, 3 main categories with 7 subcategories were defined using anchor examples. Textbox 1 provides an overview of the main categories and subcategories defined.

**Overview of the 3 main categories with a total of 7 subcategories from the analysis of interviews with experts in artificial intelligence.**

**Essential foundation**

- Awareness
- Consequences
- Data protection

**Results in the foreground**

- Performance
- Economic efficiency

**Obstacle to progress**

- Demands
- Blockade

### First Main Category: Essential Foundation

As part of the first main category (“essential foundation”), all the statements defining ethics as an essential basis for the development of AI-based applications in medicine were summarized.

#### Subcategory 1: Awareness

The first subcategory, “awareness,” highlights the relevance of ethics in development because of the potential dangers and consequences associated with AI:

Because AI is a sharp weapon, [unintelligible] it can be sharpened arbitrarily. But it must be used wisely. And I think one of the biggest difficulties is to anticipate, what does it actually mean when we develop this? [...] this anticipatory ethical question is extremely difficult.

E1; quote A.1

This subcategory emphasizes the importance of developers being cognizant of the potential uses and challenges that may arise with the subsequent implementation of AI-based applications in medical settings. An additional perspective further reinforces this view:

If we develop something, we always think the application will be used as anticipated in the clinical setting. But we can never be sure, and developers need to be aware of this.

E5; quote A.2

#### Subcategory 2: Consequences

The second subcategory “consequences,” further emphasizes the importance of ethics in practical development and an associated awareness to prevent consequences such as biases in the data or other potential forms of discrimination from being incorporated into the application:

I think everyone working with AI, especially the field of medicine or [unintelligible], should think of potential consequences involved with it. This does not only include the development teams or companies, but rather anyone.

E4; quote A.3

Although the previous quote offers a broad view of the ethical considerations in AI for medicine, the next quote from a different expert highlights specific concerns, such as bias and its potential harm to patients:

Yes, well, ethics is super important. [...] Well, when we talk about this bias, when we talk about these false negatives, it’s very important. [...] I am mostly afraid bias. Bias could really harm patients with potentially fatal outcomes. To limit the risk of any bias, we have ongoing discussions in the team.

E5; quote A.4

#### Subcategory 3: Data Protection

The importance of ethics is also highlighted in terms of the general use of human data in the development of AI-based applications, thereby forming the foundation of the third subcategory:

Well, we actually have this discussion all the time. We at [...] have an ethics working group, for ethical processing and also [unintelligible] and equality. These aspects are always there, especially when you are working with data and people, [unintelligible] data generated by people.

E4; quote A.5

### Second Main Category: Results in the Foreground

In the context of the second main category, all statements from the experts are summarized, in which the “Results are in the foreground” of the development of AI-based algorithms.

#### Subcategory 1: Performance

The following quote from the analysis of the third expert interview reflects the result-oriented nature of the development of AI-based applications in medicine, which underlies the formation of the first subcategory:

For me insofar, and I also indirectly deal with it [ethics], but for me it does not represent the first thing. So, if it’s for me, let us say, I want to set up a system first, then it’s also about, I want to set up the system. Ethical aspects do not play a role for me. [...] sounds mean now, but when an IT specialist first trains his models, it’s just about, as banal as it sounds, it’s just about achieving good performance first.

E3; quote B.1

This result and performance-driven perspective was echoed by another expert, who highlighted the competitive nature of AI development:

But I also believe that there are, let me say, more important things than ethics. Especially with the increased interest in AI, the competition is hard. [...] Developers as well as the applications do need to perform well.

E2; quote B.2

These statements collectively underscore a tendency within the industry to prioritize performance metrics, which may occasionally overshadow ethical considerations in the drive to advance and remain competitive in the rapidly evolving AI sector.

#### Subcategory 2: Economic Efficiency

The subordinate significance of ethics in performance is also clarified by the following statement in the second subcategory:

I think companies that are in competition, even if they don’t mean it badly, still have the market economic pressure to deliver results, and this can certainly also lead to losing sight of maintaining some ethical boundaries that one would better keep a careful eye on.

E6; quote B.3

This sentiment is reiterated by another expert who highlights the financial imperatives driving company behavior:

In the end, earning money and making a profit is important to anyone being paid by companies. [...] This might be different in academia, like research, but we all need to focus on creating a product that does financially well, and not trying to be ethically correct.

Interview E2; quote B.4

These perspectives elucidate the conflict that experts perceive between economic efficiency and ethical conduct in the development of AI-based medical applications.

### Third Main Category: Obstacle to Progress

The third main category summarizes statements from experts who view ethics as an “obstacle to (technological) progress.”

#### Subcategory 1: Demands

As part of the first subcategory, the “Demands” of ethics are viewed as potential barriers that can stand in the way of AI technology and the technological progress of AI-based applications in medicine:

I always find it a bit difficult to draw this line between these ethical demands and the limits that then really stand in the way of technology and progress.

E6; quote C.1

#### Subcategory 2: Blockade

The perception that ethics can not only hinder current development but also impede future progress in AI forms the basis of the “Blockade” subcategory. This is exemplified by the following statement:

Please stop bothering me on the topic of ethics in AI. It blocks at all corners and edges. [...] Yes, but if I don’t start, how should someone else continue in ten, 20 years so that something comes out of it?

E7; quote C.2

The aforementioned quote illustrates a dismissive attitude toward ethics as part of the development process of AI-based applications in medicine and thus clarifies the assessment of ethics as an obstacle to (technological) progress. This perspective was reinforced by an additional quote from another expert:

I have no doubt that ethics is important, but it does not help the technological progress of AI. [...] Ethics can really prevent any meaningful advancement.

E6; quote C.3

Together, these quotes highlight a critical perspective within the AI development community, where ethical concerns, although important, are sometimes seen as obstructions to both immediate technological development and long-term innovation in AI.

## Discussion

### Principal Findings

The results of the qualitative content analysis revealed a nuanced spectrum of expert opinions regarding the role of ethics in AI development for medical applications. Initially, in the “essential foundation” category, a consensus was observed among experts (eg, E1 and E5) on the foundational importance of ethics in AI development. This consensus on the foundational role of ethics is based on an understanding of AI’s potential risks and consequences of AI, as exemplified by the anticipatory ethical questions posed by E1 (quote A.1) and the emphasis on uncertainty in application outcomes noted by E5 (quote A.2).

Within the “results in the foreground” category, a shift in perspective becomes apparent. Experts, such as E3 and E2, express views that prioritize performance and competitive outcomes over ethical considerations (quotes B.1 and B.2). This shift suggests a conflict between ethical integrity and market-driven objectives, with the latter often taking precedence in the fast-paced competitive landscape of AI development.

In the “obstacle to progress” category, the tension between ethical demands and technological advancement is further articulated. Expert E6, for instance, acknowledged the difficulty of reconciling ethical demands with the limits imposed on technology and progress (quote C.1). This sentiment is echoed by expert E7, who expresses frustration with ethics perceived as a blockade of development (quote C.2). These perspectives underscore a critical view within the AI development community, where ethical concerns, although recognized as important, are sometimes seen as obstacles to immediate technological development and long-term innovation.

This variety of opinions, ranging from viewing ethics as foundational to considering them as impediments, reflects the complex and multifaceted nature of AI development in medicine. This demonstrates that although there is a general recognition of the importance of ethics, the extent to which it is prioritized differs significantly among experts. This diversity highlights the challenges in balancing ethical considerations with other developmental goals, such as performance optimization, economic viability, and technological innovation.

The analysis of the expert interviews identified 3 critical themes: first, the incompleteness of data and the far-reaching consequences associated with it; second, the renunciation of ethical requirements because of economic pressure; and third, the opinion that adhering to ethical standards would stand in the way of technological progress. These themes, reflecting a spectrum of perspectives from foundational importance to perceived obstacles, are explored in detail in subsequent sections, providing a deeper understanding of the multifaceted nature of ethics in AI development for medicine.

### Incompleteness of Data

Quote A.4 (section *Subcategory 2: Consequences*) refers to the relevance of biases in the data. The lack of representativeness of the data, which underlies the development of AI-based applications, has been cited as a fundamental potential bias. Although awareness of the potential consequences, such as discrimination against certain population groups, is a crucial first step, it is not enough to merely recognize the issue to avoid potentially significant consequences [38]. Therefore, active measures must be taken to prevent these biases and ensure that AI-based applications do not perpetuate or exacerbate inequalities, thereby limiting potential harm.

To mitigate bias risks, developers should adopt comprehensive strategies, such as inclusive data collection methods, algorithmic audits, thorough testing across various demographic groups, and ongoing bias monitoring throughout the AI application lifecycle. As highlighted in quote A.1, the anticipatory ethical question in AI development is “extremely difficult,” underscoring the complexity of ensuring that AI systems are ethically sound and free from biases that could lead to discrimination or harm. Interdisciplinary teams, including ethicists and representatives from diverse communities, should guide the development process to ensure that ethical considerations are at the forefront of AI development.

A potential consequence of nonrepresentative data, as highlighted in quote A.4, includes “false negatives” in medicine, which are test results that incorrectly turn out to be negative despite the presence of diagnostic features of the disease under investigation [25]. However, it is also critical to recognize that the same issue of nonrepresentativeness can lead to “false positives,” where tests incorrectly indicate the presence of a condition that is actually absent [25]. Both types of diagnostic inaccuracies have serious implications for patient care and treatment outcomes. This is further compounded by the sentiment expressed in quote A.3, where the need for everyone working with AI, especially in medicine, to consider the potential consequences of their work is emphasized, indicating a broader responsibility beyond development teams. This emphasizes the need for a comprehensive approach to diagnostic accuracy that accounts for both the presence of representative data and various factors influencing AI performance, extending beyond data representativeness [26]. Accuracy is also determined by the quality and variety of information subject to analysis from AI-based applications, including clinical, laboratory, and patient-reported data [39]. Furthermore, how AI processes and interprets this information, such as through its underlying algorithms and decision-making logic, is highly important for diagnostic accuracy [40]. There must be a match between the design purpose of the algorithm and real-world scenarios in which it is applied.

Moreover, the diagnostic accuracy of AI-based applications depends substantially on the proficiency with which health care professionals use these tools and their capacity to interpret and act on AI-generated recommendations [41]. For instance, if AI applications are used beyond their original scope without proper recalibration or validation for new populations or diseases, there is a risk of introducing errors, including false negatives and false positives [25].

False negatives in a clinical context can lead to physicians feeling a false sense of security and the diseases of patients remaining untreated for a long time [25]. Conversely, false positives can result in unnecessary treatments when a test erroneously indicates the presence of a disease, leading to significant consequences, such as unwarranted radiation exposure [25]. The psychological impact on patients, resulting from both false negatives and false positives, is a further concern that merits attention because of its effect on patient well-being and trust in medical systems.

The ethical implications of AI development, particularly when personal data are used, are highlighted in quote A.5 (section *Subcategory 3: Data Protection*). The use of training data for diagnosing specific diseases requires a careful ethical approach, particularly to understand the personal and clinical contexts from which such data are derived. This is particularly important for diseases that restrict the ability of the affected individuals to provide informed consent. Furthermore, ongoing discussions within ethics working groups about ethical processing, as mentioned in quote A.5, play a crucial role in safeguarding the dignity and rights of individuals whose data are used in these systems. Therefore, developers must recognize the sensitivity of medical data and the need for ethical considerations to be integrated from the outset of AI development for medical applications. Such early integration of ethics serves not only to enhance the accuracy and reliability of AI tools but also to safeguard the dignity and rights of individuals whose data are used in these systems.

### Economic Pressure

The quotes from the second main category “results in the foreground” suggest that although the interviewed experts are aware of the relevance of ethics in the development of AI-based applications, it is in conflict with their own or demanded result orientation. A possible reason for the experts’ assessment is mentioned in quote B.3 (section *Subcategory 2: Economic Efficiency*). The profitability of AI developing companies is cited as one of the reasons why ethics is subordinate to the results in practice. Companies’ economic success pressure is decisive for the success pressure of all the employees involved in development. This conflict is further illustrated in quote B.2, where an expert highlights the competitive nature of AI development, suggesting that there are “more important things than ethics” in the context of existing competition. This perspective underscores the challenge of balancing ethical considerations with the need for AI applications to perform well in competitive markets.

As quote B.1 (section *Subcategory 1: Performance*) illustrates, the best possible performance is the focus of the development. Ethics indirectly plays a role here; quote B.3 implies, in this sense, the possibility of crossing “ethical boundaries” in favor of profitability. In addition to the deliberate crossing of boundaries, this statement also implies the possibility of unconscious disregard for ethics in the development of AI-based applications. The subordinate role of ethics in profitability in development and the associated noncompliance with potential boundaries is particularly severe, as the field of application is medicine. The sentiment of economic pressure overshadowing ethical considerations is also echoed in quote B.4, in which an expert states the importance of focusing on creating a product that is financially well, often at the expense of being ethically correct.

In addition to the relevance of ethics in relation to the use of human data and the potential consequences of a lack of representativeness, patient safety should always be at the center of the development of medical products and technologies. An excessive focus on the profitability of an application can lead to the marketing of immature or faulty products, which threaten patient welfare. Furthermore, as highlighted in quote B.3, the pursuit of profitability can sometimes lead developers to overlooking ethical boundaries, potentially resulting in products that have not been thoroughly evaluated for ethical considerations and patient safety. In addition to a direct threat to patient welfare and safety, a high susceptibility to error can also lead to rejection by users and a potentially irretrievable loss of trust [42].

### Obstacle to Progress

Although the second main category cites result orientation because of economic pressure as a reason for the subordination of ethics, the third main category summarizes statements that view ethics as an “obstacle to progress.” The statements of experts in this category clearly show a rejection of ethics because of various demands and boundaries that are perceived as obstacles to the development of AI-based applications. Although no specific reasons for this assessment are provided, based on the knowledge of the steps relevant to development, it can be assumed that the statements primarily refer to regulations and requirements in the sense of a necessary positive vote by ethics committees. For data collection, use, or evaluation in the context of developing AI-based applications, compliance with certain boundaries and regulations is indispensable, not only in the medical context. However, this essential compliance is sometimes perceived by experts as a balancing act, where meeting ethical demands can create challenges in advancing AI technology (quote C.1).

These boundaries and regulations serve to protect the participants and their data. If patient data are to be used, a positive vote from an ethics committee that certifies the safety of patients and their data is necessary to begin with the respective research and data use. As ethics committees’ decisions can be time intensive depending on the type of planned research or data use and often require corrections on the part of the applicants, it is assumed that the necessity of a positive vote is one of the reasons that is viewed as an obstacle to progress. Furthermore, as highlighted in quote C.2, frustration with ethics being viewed as a blockade is evident: “Please stop bothering me on the topic of ethics in AI. It blocks at all corners and edges,” illustrating the tension between the desire for rapid AI development and the need for ethical oversight. Although it can be assumed that AI-based applications would be developed faster if no vote from an ethics committee was necessary and patient data could be used directly, the resulting consequences for patients and citizens (think of the insurance industry) at least require critical evaluation.

Furthermore, although the need for a positive vote by an ethics committee can be anticipated as a perceived obstacle to progress in the development of AI by experts, it is also important to consider ongoing regulatory efforts, such as the proposed “Artificial Intelligence Act” by the European Parliament [43]. This regulation aims to harmonize rules on AI across the European Union, focusing on human-centric and trustworthy AI. The Act emphasizes the protection of health, safety, fundamental rights, and environmental concerns from potential harm caused by AI systems. It includes specific recommendations for high-risk AI systems, such as AI-based applications for medicine, demanding transparency, accountability, and accuracy in AI applications, especially those that may significantly impact individuals’ rights and safety. The Act further acknowledges the ethical considerations in AI development and underscores the need for AI systems to adhere to robust ethical and legal standards. The regulatory requirement to adhere to ethical standards, as mandated by the Act, could further reinforce the perception of ethics and regulations being an obstacle, highlighting the tension between rapid technological advancement and the need for responsible innovation. In addition, quote C.3 conveys a sentiment shared by some experts that although ethical considerations are undeniably important, they are sometimes viewed as hindrances to meaningful AI advancement, further highlighting the complex dynamics between ethical considerations and the pursuit of technological progress in AI.

### Consequences of Neglecting Ethics in the Development of AI-Based Applications in Medicine

#### Overview

If ethics is not considered in the development process of AI-based applications, it can have far-reaching consequences for patients and physicians, such as loss of trust and erosion of patient-centered care. This section focuses on the possible consequences of neglecting ethics when developing AI-based applications in medicine. In this context, the consequences for patients and likely main users (physicians) were considered.

#### Possible Consequences for Patients

If those responsible do not consider or only marginally consider the basic ethical principles in the development process of AI-based applications, various indirect and direct consequences can occur for the patients in whom the respective AI-based applications are used. The following examples illustrate the possible consequences of not considering ethical principles in the development process of AI in medicine:

- Misdiagnosis and diminished therapy outcomes: a lack of ethical considerations in the practical development process of AI-based applications can lead to biases in the training data used for development. For example, if the applications are used for diagnosis, the lack of representativeness of the data for certain population groups or individuals can lead to a higher susceptibility to errors. The results presented by AI can lead to potentially significant consequences for patients, such as overtreatment or undertreatment, resulting in diminished therapeutic outcomes, particularly in the absence of control by users [11]. These errors, stemming from a misdiagnosis because of unrepresentative data, challenge the principle of justice by threatening equitable medical care and contravene the principle of nonmaleficence by risking patient harm through inappropriate medical procedures [24]. Moreover, susceptibility to errors may directly compromise patient outcomes, especially when undertreatment occurs because of delayed or missed treatments from false-negative results [16]. The interrelated consequences of misdiagnosis and therapy outcomes highlight the critical need for user oversight and inclusion of diverse data sets in AI development to uphold ethical standards and patient care quality.
- Loss of trust: faulty diagnoses and the possibility of AI-based applications yielding discriminatory results can significantly undermine patient trust [44]. Such erosion of trust may lead patients to view AI-based medical applications skeptically, potentially refraining from using them in their treatment. This skepticism can hinder the integration of advanced AI tools in health care, which, if more accurate than physicians’ assessments, could otherwise enhance patient outcomes. A loss of trust not only impedes technological adoption but can also indirectly challenge the principle of care, which is dedicated to optimizing patient welfare. Furthermore, patient reluctance to embrace AI solutions may inadvertently perpetuate inequalities in health care, particularly if AI facilitates more effective and efficient clinical practice. The reluctance to use AI technologies could result in disparity in care quality, as physicians may be limited in their capabilities without AI support, ultimately affecting the standard of care provided. Moreover, an erosion or lack of trust in AI because of missing ethical oversight in development could extend to the physician-patient relationship and the overall health care sector. Moreover, an erosion or lack of trust in AI because of missing ethical oversight in development could extend to the physician-patient relationship and the overall health care sector [45]. This could lead to a general skepticism toward medical advice and a hesitation to participate in newer forms of treatment, potentially reverting to more traditional but less efficient methods. The physician-patient relationship is foundational to effective health care, as it relies on mutual trust and the belief that the best possible treatment options are being used, including ethically developed AI applications.
- Data misuse: a lack of consideration of ethics in the development of AI-based applications can lead to violations of existing data protection laws and misuse of patient data [46]. Patients who provide their data for research purposes and for the development of new applications in medicine must be able to rely on careful and legally compliant handling of their data, particularly in terms of informed consent and cybersecurity. Given the lack of traceability, informed consent is crucial, as patients must have a clear understanding of how their data will be used and the ability to consent to specific uses. This is of particular importance because health-related data include personal and sensitive information about patients. Ignoring existing regulations and ethical principles can result in highly sensitive patient data becoming accessible to companies, organizations, or individuals without consent [46]. This could have far-reaching consequences such as compromising patient privacy, enabling identity theft, or even affecting the broader integrity of medical research and public trust in the health care system. Similarly, robust cybersecurity measures are essential to protect sensitive health information from unauthorized access and breaches. Failure to implement such measures can lead to the exposure of personal health data, resulting in a loss of patient trust, potential harm, and a violation of the autonomy of patients if they lose control over their own data.
- Erosion of patient-centered care: the exclusion of patient values and preferences during the development of AI-based medical applications can have profound consequences. When AI systems are designed without a thorough understanding of patient autonomy, self-determination, and individual health goals, there is a risk of eroding the essence of patient-centered care [47]. AI recommendations that do not account for these personal factors might lead to a mechanical and less social approach to health care that could disregard the nuanced needs and desires of patients. For example, if AI tools are optimized solely for clinical efficiency without considering patient comfort and personal treatment preferences, they may suggest interventions that patients find unacceptable or intrusive. This misalignment can result in decreased adherence to treatment plans, loss of trust in the physician-patient relationship, and diminished health outcomes [48]. Given the importance of autonomy in the physician-patient relationship and patient care in general, AI-based applications should be designed to support a shared decision-making model in which AI assists the therapeutic process rather than diminishing it. This would ensure that AI acts as an aid rather than a replacement for the human element in health care, empowering patients to be active participants in their treatment decisions rather than passive recipients of care.

#### Potential Consequences for Physicians

In addition to the significant consequences for patients, the lack of ethical consideration in the development process of AI-based applications in medicine can also lead to equally relevant impacts on anticipated primary users of the technology. Although the following examples primarily aim to illustrate the direct consequences for physicians, they also indirectly affect the patients being treated:

- Loss of credibility: potential errors in diagnosis or treatment recommendations resulting from inadequately trained AI applications can also significantly influence the societal image of the medical profession and its associated credibility [49]. Assuming that physicians continue to serve as the link between technology and patients, erroneous decisions based on the use of AI in medicine can be directly associated with the decision-making abilities of physicians, which can negatively impact their credibility and trust in the medical community [44]. Knowledge about the potential for discrimination of certain population groups by AI-based applications, which do not consider ethical guidelines in their development, can further shake patients’ beliefs that physicians guarantee equal treatment for all. Because a patient’s medical treatment often appears nontransparent and incomprehensible, the credibility of the medical community is an essential prerequisite for the physician-patient relationship [49].
- Rejection: the lack of consideration of ethics in the development of AI-based applications for use in a clinical context can lead to both indirect (eg, because of the consequences of incorrect diagnoses) and direct (eg, because of the lack of consideration of ethical principles) rejection of the technology by physicians. The rejection of AI-based applications can significantly impact the quality of medical care and the technological progress in medicine. Without the acceptance and trust of prospective primary users of the technology, the widespread use of AI-based applications in medicine is unlikely, as economic incentives for development are lacking. A rejecting attitude on the part of physicians can in this context also negatively impact future medical care quality considering the expected advantages of using AI in medicine [46].
- Legal consequences: the use of AI-based applications developed without considering ethical principles can lead to various legal consequences for users [50]. In addition to consequences based on state legislation and jurisprudence, professional legal consequences for physicians are also conceivable when using AI-based applications without considering ethical principles, as they form the basis of medical action. Besides the direct legal implications for physicians, health care organizations, such as hospitals, clinics, or research institutions, may also be subject to significant responsibilities and potential liabilities when deploying AI-based applications that may not fully align with ethical and regulatory standards. In the case of erroneous AI decisions, which directly or indirectly result in diminished patient outcomes, the question of legal liability often remains unanswered [51]. As AI-based applications in medicine are likely to continue to be used and developed in a supportive role, it is assumed that the final decision-making and treatment recommendations will remain the responsibility of physicians. Thus, physicians not only act as a link between technologies and patients but also play a central role in adhering to ethical principles in medical care. Against this background, the use of AI-based applications in medicine developed without considering ethics can have legal consequences for both developers and users. In addition to the legal consequences of erroneous medical treatments, the use of AI-based applications without considering ethical principles also raises questions regarding the liability for violation of existing data protection and equal treatment laws [51]. In particular, failure to comply with data protection laws can compound these legal issues. Violations of patients’ privacy rights through the mishandling of sensitive patient data, whether because of inadequate security measures, hacks, or unauthorized data sharing, may subject various entities, such as hospitals, clinics, research institutions, AI technology developers, and users to significant legal liability [50]. These data breaches not only compromise patient confidentiality but also could lead to a risk of regulatory sanctions for the involved entities, including substantial fines and potentially the loss of professional licenses. Therefore, AI development processes should incorporate robust data protection protocols to prevent legal repercussions and consequences for both patients and physicians. Adherence to ethical and legal standards should not merely be a regulatory requirement but a fundamental component of responsible and trustworthy health care innovation, vital for maintaining the integrity of patient care and the broader medical profession.

### Limitations

This study’s exploration of expert perspectives on ethics in AI development for medical applications, although insightful, encounters several limitations that are important to acknowledge. First, the geographical focus of the study was confined to Germany, potentially limiting the applicability of its findings to a global context in which cultural, legal, and ethical norms may vary. The selection of experts, although experienced in the development of AI-based applications in medicine, represents a relatively small and specific segment of the broader field. Moreover, the focus of the study, predominantly on experts with technical backgrounds in the development of AI-based applications, may lead to a narrowed perspective, given the lack of input from ethical professionals. Furthermore, the subjective nature of expert interviews should be considered because the responses are influenced by each expert’s personal experiences and potential biases, which may not comprehensively represent the spectrum of views in the field.

Methodologically, the study’s qualitative approach and reliance on secondary analysis of expert interviews inherently limits the generalizability of the results. Interpretations may be influenced by the research team’s perspectives, and certain nuances in experts’ statements may be overlooked. Although this study presents a secondary analysis of existing data, it is important to recognize the possibility of confirmation and selection bias during the initial data collection phase. The research methodology used could have unintentionally emphasized certain themes or perspectives, potentially aligning with the original researchers’ preconceived notions or expectations. In addition, because of the limited number of experts included in the analysis and incomplete data saturation in some subcategories, certain aspects may not have been fully explored.

Furthermore, the findings of this study reflect a specific point in time in a rapidly evolving field. Therefore, the perspectives and opinions of experts may change as new developments, regulations, and ethical guidelines emerge. Although substantial, the focus on the development of AI-based applications in medicine does not encompass the entire spectrum of AI applications within the health care sector, excluding administrative and operational uses. Language and translation limitations may also have affected the study, as the original German interviews were translated into the English language. The subtle nuances of language and cultural context might be lost or misinterpreted in this translation process.

To address these limitations and enrich future research in this area, it is recommended that subsequent studies incorporate a broader and more diverse pool of experts, including professionals from ethical, legal, and patient advocacy backgrounds. Expanding the geographical scope to include experts from various cultural and legal contexts would also provide a global perspective on the ethical implications of developing AI-based applications for medicine. Methodologically, integrating both qualitative and quantitative approaches could offer a more comprehensive view, although ongoing research is required, considering the rapid advancements in AI and evolving ethical standards. By expanding the scope and methodology of future studies, a more nuanced and representative exploration of the ethical landscape of AI development for medical applications can be achieved.

### Summary and Outlook

This study explored the importance of ethics in the development of AI-based medical applications by analyzing interviews with experts in the field of AI development. There was substantial variance in the assessment of the importance of ethics in the development of the AI-based applications. Although some of the interviewed experts classified ethics as an essential basis for development, others focused on good performance or economic efficiency. The results of the qualitative analysis also suggest that ethics is seen by some experts as an obstacle to progress, implying that it will be given little importance in the further development of AI-based applications. In addition to the subsequent discussion of the content analysis results, a particular focus was placed on the consequences that could arise from the lack of ethical considerations in the development of AI-based applications in medicine.

Although the results do not allow for generalization, because of the number of interviewees and the selected qualitative research method not meeting representative demands, the statements of the interviewed experts should be seen as an essential basis for further research and discussions because of recurring motives and new insights. A lack of ethical considerations in the development of AI-based applications can have significant consequences for patients. In addition to the danger of misconduct (eg, because of a lack of representativeness of the data sets used for development), a lack of consideration of ethical principles in the development of AI-based applications can also lead to a loss of trust from patients and potentially diminished therapy outcomes. When considering the possible impacts on physicians, the lack of consideration of ethics in the development process can lead to loss of credibility and rejection of technology.

Owing to technological progress in the field of AI, further reinforced, for example, by the development and broad availability of AI-based chat applications such as ChatGPT, there has been ongoing effort to develop guidelines and laws to guide the development and use of AI. Although such regulatory efforts, such as the “Artificial Intelligence Act” for harmonized rules on AI from the European Parliament, aim to provide a comprehensive regulatory framework and guideline for the development and use of AI, there is ongoing criticism and discussion about the adequacy and effectiveness of these guidelines in the rapidly evolving field of AI. In this context, it is important to emphasize that the sole availability of guidelines and laws does not ensure compliance. Therefore, although guidelines and laws are important to guide the development and use of AI, especially in the field of medicine, and when dealing with sensitive patient data, more work needs to be done to ensure compliance.

Moreover, the question arises as to whether mere adherence to these guidelines and laws is sufficient for the development of ethical AI. Guidelines often provide a baseline for legal compliance, but ethical AI development demands a deeper and more nuanced understanding and application of ethical principles. Ethical AI goes beyond legal requirements to encompass ethical principles, such as respect for autonomy or justice in its algorithms, data handling, and decision-making processes. This requires continuous ethical assessment and reflection throughout the lifecycle of AI-based applications, from development to deployment, and beyond. Consequently, although following established guidelines is an important step in the development of AI, it is not the endpoint. Developers and users of AI-based applications in medicine need to engage in an ongoing dialog with diverse stakeholders such as ethicists, patients, and the broader community to anticipate, identify, and address emerging ethical challenges. This approach ensures that the development of AI is not just about complying with regulations but is intrinsically driven by a commitment to ethical responsibility and the betterment of patient care.

Furthermore, possible reasons for noncompliance with potential guidelines and low prioritization of ethics, such as the need for economic efficiency, should be critically examined. This includes assessing perspectives that view ethics as an obstacle to progress, as noted by some participating experts. Such critical evaluation is vital for ensuring the ethical development of AI-based applications, particularly in the field of medicine. Ethical considerations are fundamental to every approval process for AI-based applications to ensure the best possible and equal medical care for patients. Therefore, physicians should critically question the use of AI-based applications in the clinical context. In this regard, there needs to be a sufficient availability of opportunities to acquire further competencies to promote an understanding of technology and the related relevance of ethics. Only in this manner can the safety and best possible treatment of patients be ensured, as well as medical and technological progress, through AI.

## Abbreviations

AI: artificial intelligence
CDSS: Clinical Decision Support Systems
ML: machine learning
